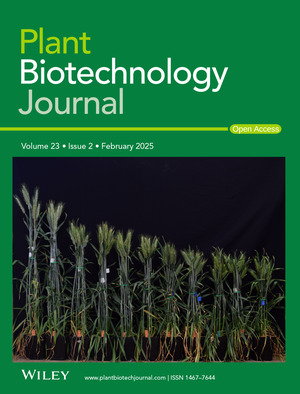# Issue Information

**DOI:** 10.1111/pbi.14388

**Published:** 2025-01-27

## Abstract

Front cover image:

Fine‐tuning triticale plant height using CRISPR mutations on the miR172 binding sites of *AP2L*. Cover illustration refers to the article published in this issue (Zhang et al., pp. 333–335).